# A Novel c.91dupG* JAG1* Gene Mutation Is Associated with Early Onset and Severe Alagille Syndrome

**DOI:** 10.1155/2018/1369413

**Published:** 2018-06-25

**Authors:** Alejandra del Pilar Reyes-de la Rosa, Gustavo Varela-Fascinetto, Constanza García-Delgado, Edgar Ricardo Vázquez-Martínez, Pedro Valencia-Mayoral, Marco Cerbón, Verónica Fabiola Morán-Barroso

**Affiliations:** ^1^Department of Genetics, Hospital Infantil de México Federico Gómez, Mexico City, Mexico; ^2^Department of Transplantation, Hospital Infantil de México Federico Gómez, Mexico City, Mexico; ^3^Unidad de Investigación en Reproducción Humana, Instituto Nacional de Perinatología Isidro Espinosa de los Reyes-Facultad de Química, Universidad Nacional Autónoma de México, Mexico City, Mexico; ^4^Department of Pathology, Hospital Infantil de México Federico Gómez, Mexico City, Mexico

## Abstract

Alagille syndrome (MIM 118450) is an autosomal dominant disorder characterized by paucity of intrahepatic bile ducts, chronic cholestasis, pulmonary stenosis, butterfly-like vertebrae, posterior embryotoxon, and dysmorphic facial features. Most cases are caused by* JAG1 *gene mutations. We report the case of a 2-year-old Mexican mestizo patient with Alagille syndrome, having exhibited jaundice and cholestatic syndrome as of three weeks of age. Sequencing analysis of the* JAG1* gene revealed the novel heterozygous mutation c.91dupG that originates a truncated protein and therefore a possibly diminished activation of the Notch signaling pathway. The latter may explain the severe phenotype of the patient. Since the mutation was not identified in the parents, it was considered a* de novo* event, highlighting the importance of molecular diagnosis and genetic counseling. In conclusion, this report widens the spectrum of* JAG1* gene mutations associated with Alagille syndrome.

## 1. Introduction

Alagille syndrome (ALGS1; MIM 118450) is an autosomal dominant disorder with prevalence of 1:70,000-100,000. It presents a broad spectrum of clinical manifestations, characterized by paucity of intrahepatic bile ducts, chronic cholestasis, pulmonary stenosis, butterfly-like vertebrae, posterior embryotoxon, and a dysmorphic face characterized by a broad forehead, deep-set eyes, a bulbous nose, and a small pointed mandible [1, 2].

In 97% of the cases, ALGS1 is due to* JAG1 *mutations (MIM 601920). The* JAG1* gene is in 20p12.2 and encodes for JAGGED1, a 1218 amino acid protein that participates in the Notch signaling pathway as a ligand. This pathway is involved in the transcription of genes for cell fate and differentiation.* NOTCH2 *gene mutations (MIM 600275) are identified in less than 1% of ALGS patients (ALGS2). [3, 4]

ALGS1 is established when three of the five major clinical features are diagnosed and can be confirmed by direct sequencing of* JAG1* [5]. Next-generation sequencing analysis, including either genome or exome sequences, has been recommended for the molecular diagnosis of neonatal or infantile intrahepatic cholestasis [6]. Our institution is a national reference center for the diagnosis and management of ALGS patients, in which novel and known* JAG1* mutations have been identified in Mexican mestizo patients [7]. Here we describe a patient having a new* JAG1 *mutation associated with early onset and severe Alagille syndrome.

## 2. Case Presentation

**Figure 1:**
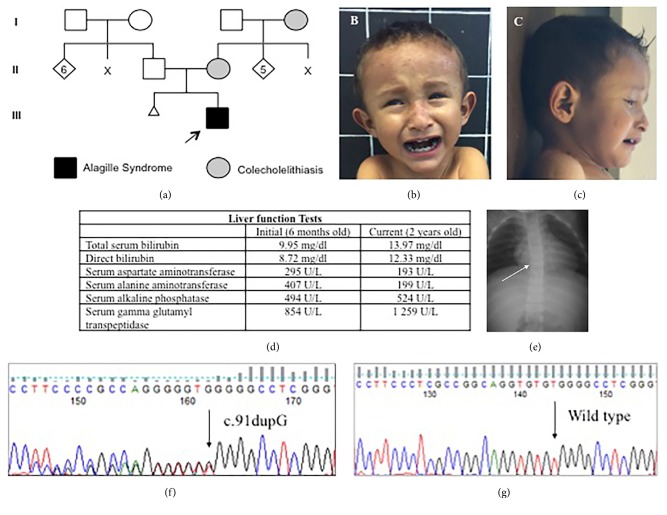
(a) Pedigree of the family of the proband. (b) Facial clinical characteristics of the proband. Note the triangular face, wide forehead, and prominent chin. (c) Deep-set eyes. (d) Altered liver function analysis. (e) X-ray analysis showing butterfly-like dorsal vertebra (white arrow). (f) Capillary sequencing analysis demonstrating the mutation in the proband. (g) Wild-type sequence in one of the parents.

The proband is a 2-year and 7-month-old Mexican mestizo male (Figure 1(a), individual III.2). He was referred at 5 months of age due to cholestatic syndrome characterized by jaundice and pale stools, which began as of the third week of life. He is the only child of a young, apparently healthy, and unrelated couple.

The pregnancy lasted 39 weeks and was complicated by maternal cholelithiasis at the fourth month. Antibiotics were indicated for repetitive urinary infections as of the fifth month. Delivery was carried out by cesarean section due to fetal distress. The newborn's weight was 2200 g (P<5), height was 48 cm (P<5), and Apgar score was 8/9.

The cognitive development of the patient is normal. At nine months of age, his weight was 6 kg (P<5), height was 64 cm (P<5), and head circumference was 44.5 cm (P25-50). He manifested generalized jaundice, dry skin, and an anterior fontanelle that had not yet closed. He had sparse eyebrows, a broad forehead, deep-set eyes, a triangular face, prominent ears, a heart murmur, hepatomegaly, and hypotrophic limbs (Figures 1(b) and 1(c)). His liver function tests were abnormal (Figure 1(d)) and an abdominal ultrasound analysis demonstrated generalized thickening of the biliary tract. The X-ray analysis showed a butterfly-like image in several dorsal vertebrae (Figure 1(e)). Right and left pulmonary hypoplasia were diagnosed by echocardiogram analysis. A magnetic resonance image analysis at the age of 1 year and 2 months displayed widening of the subarachnoid space and bilateral subarachnoid cysts in the temporal fossa. The optic nerve in both eyes was normal. At 2 years and 2 months of age, he developed xanthomata in both elbows and in his knuckles, and at 2 years and 4 months of age, posterior embryotoxon was diagnosed. A hepatic biopsy detected intracytoplasmic cholestasis and the absence of interlobular conducts.

*JAG1* gene molecular analysis was conducted with institutional approval and after obtaining informed consent by second-generation sequencing in a private laboratory (Nanolab, Mexico City, Mexico) from blood samples, revealing the c.91dupG variant in a heterozygous state. This causes a frameshift mutation and a premature stop codon at amino acid number 72 of the JAGGED1 protein (Figure 1(f)). Capillary sequencing was carried out in our laboratory and confirmed the presence of the mutation, which was not found in the parents (Figure 1(g)).

## 3. Discussion

Alagille syndrome is an infrequent disease requiring a multidisciplinary team of medical specialists for its management [5, 6]. In the patient currently under study, the onset of Alagille syndrome was at an early age with severe clinical characteristics, including cholestatic syndrome and xanthomata.* JAG1* gene molecular analysis revealed the previously unreported c.91dupG variant in a heterozygous state, which produces a truncated JAGGED1 protein and probably a diminished function of the Notch signaling pathway. However, additional studies are required to explore its effect on the protein and signaling level.

Mouzaki et al. reported that some symptoms are deemed indicators of a bad prognosis, such as high total bilirubin levels between 12 and 24 months of age, liver fibrosis, and xanthomata [8]. The present patient had two of these predictors, high total bilirubin and xanthomata, and required a liver transplant.

ALGS1 is considered to have a wide spectrum of clinical variabilities with no strong phenotype-genotype correlation. In some cases, as found herein, there can be early onset and severe symptoms [6, 8]. As the mutation of the patient in question was not identified in his parents, it was considered a* de novo *event, which occurs in up to 60% of ALGS1 cases. However, a germline mosaicism in one of the parents cannot be discarded [7–9]. Both the latter scenario and a mutation are important aspects for genetic counseling. The current report widens the spectrum of* JAG1* gene mutations associated with Alagille syndrome.

## Abbreviations

ALGS: Alagille syndrome.
